# Continuity conditions for Q-Bézier curves of degree *n*

**DOI:** 10.1186/s13660-017-1390-3

**Published:** 2017-05-16

**Authors:** Gang Hu, Cuicui Bo, Xinqiang Qin

**Affiliations:** Department of Applied Mathematics, Xi’an University of Technology, Xi’an, 710054 P.R. China

**Keywords:** 65D07, 65D10, 65D17, 65D18, 68U05, 68U07, Q-Bézier curve, shape parameter, geometric continuity, curve design

## Abstract

As a new method of representing curves, Q-Bézier curves not only exhibit the beneficial properties of Bézier curves but also allow effective shape adjustment by changing multiple shape parameters. In order to resolve the problem of not being able to construct complex curves using a single curve, we study the geometric continuity conditions for Q-Bézier curves of degree *n*. Following the analysis of basis functions and terminal properties of Q-Bézier curves of degree *n*, the continuity conditions of $\mathrm{G}^{1}$ and $\mathrm{G}^{2}$ between two adjacent Q-Bézier curves are proposed. In addition, we discuss the specific steps of smooth continuity for Q-Bézier curves and analyze the influence rules of shape parameters for Q-Bézier curves. The modeling examples show that the proposed method is effective and easy to achieve, making it useful for constructing complex curves for engineering design.

## Introduction

Parametric curves are not only an important research area in CAD/CAM, but also a powerful tool for shape design. Classical Bézier curves are constructed using Bernstein basis functions, which have a simple structure and are easy to use. The latter have already become one of the most important methods in the CAD/CAM field. However, the shapes of Bézier curves are only determined by the control points, which causes issues for engineering design. In order to overcome this shortcoming, rational Bézier curves can be used, and their shapes can be modified or adjusted by introducing weight factors without changing their control points. However, the introduction of rational fractions produces some other issues, such as complex calculations, cumbersome integrals, and repetitive differentiation [1, 2].

In order to maintain the advantages of the Bézier method and enhance the shape adjustability of the curves, scholars have constructed many non-rational Bézier curves with shape parameters [3–19]. A set of generalized Bernstein basis functions was proposed in [20], constructing a type of Q-Bézier curve with multiple shape parameters. These generalized Bernstein basis functions inherited many of the beneficial properties of Bernstein basis functions, and the Q-Bézier curves also inherited many beneficial properties of Bézier curves. Moreover, the Q-Bézier curve also had flexible shape adjustability, with the shape of the curve being easily modified by changing shape parameters, thus creating complex curves with more degrees of freedom. Therefore, Q-Bézier curves can be widely used in various CAD/CAM systems.

As the Q-Bézier curve is a type of polynomial curve, it has inevitably inherited the instability that calculations of high-order polynomials suffer from. Consequently, the control of the polygon on the curve will be weakened when the degree of the Q-Bézier curve is too high; by contrast, a lower degree cannot express a complex curve any better. Based on this, in order to describe a Q-Bézier curve with more extensive applications in the CAD/CAM field, in this paper we derive the geometric continuity conditions between two adjacent Q-Bézier curves by analyzing the basis functions and terminal properties of the Q-Bézier curve. The resultant curves are flexible enough to be used in a wide variety of engineering design applications.

## The family of Q-Bézier curves

### Generalized Bernstein basis functions

#### Definition 1

For any natural number *n* ($n \ge 2$) and *n* arbitrary real values of $\lambda_{i}$ ($i = 1,2, \ldots,n$), the following polynomial functions in *t*

1 $$ \textstyle\begin{cases} b_{0,n}(t) = (1 - t)^{n}(1 - \lambda_{1}t), \\ b_{i,n}(t) = t^{i}(1 - t)^{n - i} ( ( {\scriptsize\begin{matrix}{} n \cr i \end{matrix}} ) + \lambda_{i} - \lambda_{i}t - \lambda_{i + 1}t ), \quad i = 1,2, \ldots, [ \frac{n}{2} ] - 1, \\ b_{ [ \frac{n}{2} ],n}(t) = t^{ [ \frac{n}{2} ]}(1 - t)^{n - [ \frac{n}{2} ]} ( ( {\scriptsize\begin{matrix}{} n \cr [ \frac{n}{2} ] \end{matrix}} ) + \lambda_{ [ \frac{n}{2} ]} - \lambda_{ [ \frac{n}{2} ]}t + \lambda_{ [ \frac{n}{2} ] + 1}t ), \\ b_{i,n}(t) = t^{i}(1 - t)^{n - i} ( ( {\scriptsize\begin{matrix}{} n \cr i \end{matrix}} ) - \lambda_{i} + \lambda_{i}t + \lambda_{i + 1}t ), \quad i = [ \frac{n}{2} ] + 1, \ldots,n - 1, \\ b_{n,n}(t) = t^{n}(1 - \lambda_{n} + \lambda_{n}t) \end{cases} $$

are called the generalized Bernstein basis functions of degree *n*, associated with the shape parameters $\{ \lambda_{i} \}_{i = 1}^{n}$ [20], where $\lambda_{i} \in [ - ( {\scriptsize\begin{matrix}{} n \cr i \end{matrix}} ), ( {\scriptsize\begin{matrix}{} n \cr i - 1 \end{matrix}} ) ]$, $i = 1,2, \ldots, [ \frac{n}{2} ]$, $\lambda_{i} \in [ - ( {\scriptsize\begin{matrix}{} n \cr i - 1 \end{matrix}} ), ( {\scriptsize\begin{matrix}{}n \cr i \end{matrix}} ) ]$, $i = [ \frac{n}{2} ] + 1, \ldots,n$,

$$\biggl[ \frac{n}{2} \biggr] = \textstyle\begin{cases} \frac{n}{2}, & \text{if $n$ is even}, \\ \frac{n + 1}{2}, & \text{if $n$ is odd}, \end{cases}\displaystyle \quad t \in [0,1]. $$

It can be easily proved that the generalized Bernstein basis functions $b_{i,n}(t)$ of degree *n* have many properties similar to those of classical Bernstein basis functions of degree *n*, such as non-negativity, partition of unity, symmetry, etc. [20]. Specifically, when the shape parameters are $\lambda_{i} = 0$ ($i = 1,2, \ldots,n$), the generalized Bernstein basis functions of degree *n* degenerate into the classical Bernstein basis functions of degree *n*.

#### Theorem 1

*The generalized Bernstein basis functions of degree* *n*, *as shown in* (1), *associated with the shape parameters* $\lambda_{i}$ ($i = 1,2, \ldots,n$), *are linearly independent*.

#### Proof

First, using degree elevation of the Bernstein basis functions of degree *n* and combining with (1), we can obtain the following equations to convert classical Bernstein basis functions to generalized Bernstein basis functions:

2 $$ \textstyle\begin{cases} b_{0,n}(t) = B_{0,n + 1}(t) + \frac{ ( {\scriptsize\begin{matrix}{} n \cr 0 \end{matrix}} ) - \lambda_{1}}{ ( {\scriptsize\begin{matrix}{} n + 1 \cr 1 \end{matrix}} )}B_{1,n + 1}(t), \\ b_{i,n}(t) = \frac{ ( {\scriptsize\begin{matrix}{} n \cr i \end{matrix}} ) + \lambda_{i}}{ ( {\scriptsize\begin{matrix}{} n + 1 \cr i \end{matrix}} )}B_{i,n + 1}(t) + \frac{ ( {\scriptsize\begin{matrix}{} n \cr i \end{matrix}} ) - \lambda_{i + 1}}{ ( {\scriptsize\begin{matrix}{} n + 1 \cr i + 1 \end{matrix}} )}B_{i + 1,n + 1}(t), \quad i = 1,2, \ldots, [ \frac{n}{2} ] - 1, \\ b_{ [ \frac{n}{2} ],n}(t) = \frac{ ( {\scriptsize\begin{matrix}{} n \cr [ \frac{n}{2} ] \end{matrix}} ) + \lambda_{ [ \frac{n}{2} ]}}{ ( {\scriptsize\begin{matrix}{} n + 1 \cr [ \frac{n}{2} ] \end{matrix}} )}B_{ [ \frac{n}{2} ],n + 1}(t) + \frac{ ( {\scriptsize\begin{matrix}{} n \cr [ \frac{n}{2} ] \end{matrix}} ) + \lambda_{ [ \frac{n}{2} ] + 1}}{ ( {\scriptsize\begin{matrix}{} n + 1 \cr [ \frac{n}{2} ] + 1 \end{matrix}} )}B_{ [ \frac{n}{2} ] + 1,n + 1}(t), \\ b_{i,n}(t) = \frac{ ( {\scriptsize\begin{matrix}{} n \cr i \end{matrix}} ) - \lambda_{i}}{ ( {\scriptsize\begin{matrix}{} n + 1 \cr i \end{matrix}} )}B_{i,n + 1}(t) + \frac{ ( {\scriptsize\begin{matrix}{} n \cr i \end{matrix}} ) + \lambda_{i + 1}}{ ( {\scriptsize\begin{matrix}{} n + 1 \cr i + 1 \end{matrix}} )}B_{i + 1,n + 1}(t), \quad i = [ \frac{n}{2} ] + 1, [ \frac{n}{2} ] + 2, \ldots,n - 1, \\ b_{n,n}(t) = \frac{ ( {\scriptsize\begin{matrix}{} n \cr n \end{matrix}} ) - \lambda_{n}}{ ( {\scriptsize\begin{matrix}{} n + 1 \cr n \end{matrix}} )}B_{n,n + 1}(t) + B_{n + 1,n + 1}(t), \end{cases} $$

where

$$B_{i,n + 1}(t) = \left ( \begin{matrix} n + 1 \\ i \end{matrix} \right ) (1 - t)^{n + 1 - i}t^{i} = \frac{(n + 1)!}{i!(n + 1 - i)!}(1 - t)^{n + 1 - i}t^{i}, \quad t \in [0,1], i = 0,1, \ldots,n + 1, $$

are the Bernstein basis functions of degree $n+1$.

Let $\sum_{i = 0}^{n} \alpha_{i}b_{i,n}(t) = 0$, where $\alpha_{i} \in R$, $i = 0,1, \ldots,n$. Then, according to (2), we can obtain

3 $$ 0 = \sum_{i = 0}^{n} \alpha_{i}b_{i,n}(t) = \sum_{i = 0}^{n + 1} \beta_{i}B_{i,n + 1}(t), $$

where

4 $$ \textstyle\begin{cases} \beta_{0} = \alpha_{0}, \\ \beta_{i} = (1 - \zeta_{i})\alpha_{i - 1} + \zeta_{i}\alpha_{i},\quad i = 1,2, \ldots, [ \frac{n}{2} ], \\ \beta_{i} = \zeta_{i}\alpha_{i - 1} + (1 - \zeta_{i})\alpha_{i},\quad i = [ \frac{n}{2} ] + 1, [ \frac{n}{2} ] + 2, \ldots,n, \\ \beta_{n + 1} = \alpha_{n}, \end{cases} $$

where

$$\zeta_{i} = \frac{ ( {\scriptsize\begin{matrix}{} n \cr i \end{matrix}} ) + \lambda_{i}}{ ( {\scriptsize\begin{matrix}{} n + 1 \cr i \end{matrix}} )},\quad i = 1,2, \ldots,n. $$

Since the Bernstein basis functions of degree $n+1$ are linearly independent, we obtain $\beta_{i} = 0$ ($i = 0,1, \ldots,n + 1$). Thus, it is obvious that $\alpha_{i} = 0$ for $i = 0,1, \ldots,n$, meaning that $b_{i,n}(t)$ ($i = 0,1, \ldots,n$) are linearly independent. □

### Definition and properties of Q-Bézier curve

#### Definition 2

Let points $\boldsymbol{P}_{i} \in R^{d}$ ($d = 2,3$; $i = 0,1, \ldots,n$), then the polynomial curve of degree *n* associated with shape parameters $\{ \lambda_{i} \}_{i = 1}^{n}$, a so-called Q-Bézier curve, can be defined as follows [20]:

5 $$ \boldsymbol{r}(t) = \sum_{i = 0}^{n} \boldsymbol{P}_{i}b_{i,n}(t), \quad t \in [ 0,1 ], $$

where points $\boldsymbol{P}_{i}$ ($i = 0,1, \ldots,n$) are control points of the curve, $\lambda_{i} \in [ - ( {\scriptsize\begin{matrix}{} n \cr i \end{matrix}} ), ( {\scriptsize\begin{matrix}{} n \cr i - 1 \end{matrix}} ) ]$, $i = 1,2, \ldots, [ \frac{n}{2} ]$, $\lambda_{i} \in [ - ( {\scriptsize\begin{matrix}{} n \cr i - 1 \end{matrix}} ), ( {\scriptsize\begin{matrix}{} n \cr i \end{matrix}} ) ]$, and $i = [ \frac{n}{2} ] + 1, \ldots,n$, $b_{i,n}(t)$ ($i = 0,1, \ldots,n$) are the generalized Bernstein basis functions of degree *n* defined by (1). According to the definition and properties of the generalized Bernstein basis functions, it is easy to see that the Q-Bézier curve has inherited many of the characteristics of the Bézier curve, such as symmetry, convex hull property, geometric invariance, etc. In addition, the Q-Bézier curve has good shape adjustability, with the shape of the curve being determined by its own control points and shape parameters. Specifically, with shape parameters $\lambda_{i} = 0$ ($i = 1,2, \ldots,n$), the Q-Bézier curve degenerates to a classical Bézier curve.

#### Theorem 2

*The Q*-*Bézier curve* $\boldsymbol{r}(t)$ *of degree* *n* *has the following terminal properties*:

6 $$ \textstyle\begin{cases} \boldsymbol{r}(0) = \boldsymbol{P}_{0} ,\\ \boldsymbol{r}(1) = \boldsymbol{P}_{n} ,\\ \boldsymbol{r}'(0) = (n + \lambda_{1})(\boldsymbol{P}_{1} - \boldsymbol{P}_{0}) ,\\ \boldsymbol{r}'(1) = (n + \lambda_{n})(\boldsymbol{P}_{n} - \boldsymbol{P}_{n - 1}) ,\\ \boldsymbol{r}''(0) = [n(n - 1) + 2n\lambda_{1}]\boldsymbol{P}_{0} - [2n(n - 1) + 2n\lambda_{1} + 2\lambda_{2}]\boldsymbol{P}_{1} + [n(n - 1) + 2\lambda_{2}]\boldsymbol{P}_{2} ,\\ \boldsymbol{r}''(1) = [n(n - 1) + 2n\lambda_{n}]\boldsymbol{P}_{n} - [2\lambda_{n - 1} + 2n(n - 1) + 2n\lambda_{n}]\boldsymbol{P}_{n - 1} \\ \hphantom{\boldsymbol{r}''(1) =}{}+ [n(n - 1) + 2\lambda_{n - 1}]\boldsymbol{P}_{n - 2}. \end{cases} $$

#### Proof

According to (1), the generalized Bernstein basis functions $b_{i,n}(t)$ ($i = 0,1, \ldots,n$; ${n \ge 2}$) at the terminal points are

7 $$\begin{aligned}& b_{i,n}(0) = \textstyle\begin{cases} 1 & (i = 0), \\ 0 & (i \ne 0), \end{cases}\displaystyle \end{aligned}$$

8 $$\begin{aligned}& b_{i,n}(1) = \textstyle\begin{cases} 1 & (i = n), \\ 0 & (i \ne n), \end{cases}\displaystyle \end{aligned}$$

9 $$\begin{aligned}& b'_{i,n}(0) = \textstyle\begin{cases} - (n + \lambda_{1}) & (i = 0) , \\ n + \lambda_{1} & (i = 1), \\ 0 & (i = 2,3, \ldots,n), \end{cases}\displaystyle \end{aligned}$$

10 $$\begin{aligned}& b'_{i,n}(1) = \textstyle\begin{cases} - (n + \lambda_{n}) &(i = n - 1), \\ n + \lambda_{n} & (i = n) , \\ 0 & (i = 0,1, \ldots,n - 2), \end{cases}\displaystyle \end{aligned}$$

11 $$\begin{aligned}& b''_{i,n}(0) = \textstyle\begin{cases} n(n - 1) + 2\lambda_{1}n &(i = 0), \\ - [2n(n - 1) + 2n\lambda_{1} + 2\lambda_{2}] &(i = 1) ,\\ n(n - 1) + 2\lambda_{2} & (i = 2) ,\\ 0 & (i = 3,4, \ldots,n), \end{cases}\displaystyle \end{aligned}$$

12 $$\begin{aligned}& b''_{i,n}(1) = \textstyle\begin{cases} n ( n - 1 ) + 2\lambda_{n}n & (i = n), \\ - [ 2n\lambda_{n} + 2n ( n - 1 ) + 2\lambda_{n - 1} ] &(i = n - 1) ,\\ n ( n - 1 ) + 2\lambda_{n - 1} & (i = n - 2), \\ 0& (i = 0,1, \ldots,n - 3). \end{cases}\displaystyle \end{aligned}$$

For the terminal properties (7)-(12) of the basis functions, as well as the definition of the Q-Bézier curve, we can produce the terminal properties (6) of the Q-Bézier curve, thus, proving Theorem 2. □

**Figure 1:**
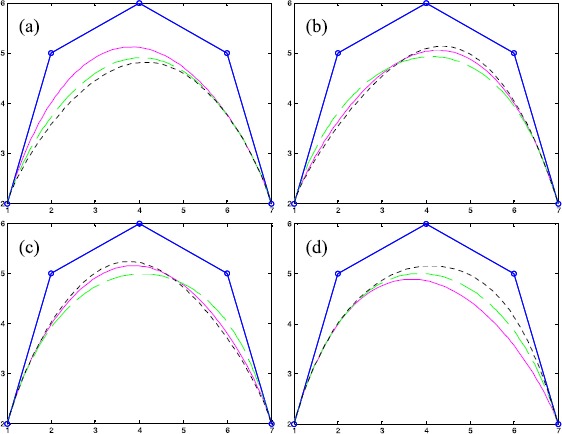
**The effect of altering the shape parameters of a Q-Bézier curve.**

Figure 1 shows the influence on the shapes of Q-Bézier curves of degree 4 by altering four parameters on the curves. Figure 1(a) shows the curves with $\lambda_{2} = 2$, $\lambda_{3} = 1$, $\lambda_{4} = - 1$, $\lambda_{1} = 1$ (solid lines), $\lambda_{1} = - 1$ (dashed lines), $\lambda_{1} = - 2$ (dotted lines). Figure 1(b) shows the curves with $\lambda_{1} = - 2$, $\lambda_{3} = 2$, $\lambda_{4} = 1$, $\lambda_{2} = 1$ (solid lines), $\lambda_{2} = - 4$ (dashed lines), $\lambda_{2} = 4$ (dotted lines). Figure 1(c) shows the curves with $\lambda_{1} = 1$, $\lambda_{2} = 3$, $\lambda_{4} = - 1$, $\lambda_{3} = 1$ (solid lines), $\lambda_{3} = - 5$ (dashed lines), $\lambda_{3} = 4$ (dotted lines). Figure 1(d) shows the curves with $\lambda_{1} = 1$, $\lambda_{2} = 1$, $\lambda_{3} = - 2$, $\lambda_{4} = - 3$ (solid lines), $\lambda_{4} = - 1$ (dashed lines), $\lambda_{4} = 1$ (dotted lines). The broken lines indicate the control polygons, and the circular points indicate control points of the curve.

## $\mathrm{G}^{1}$ and $\mathrm{G}^{2}$ smooth continuity conditions for Q-Bézier curves

Given two adjacent Q-Bézier curves $\boldsymbol{r}_{1}(t) = \sum_{i = 0}^{n} \boldsymbol{P}_{i}b_{i,n}(t)$ with control points $\boldsymbol{P}_{i}$ ($i = 0,1, \ldots,n$) and $\boldsymbol{r}_{2}(t) = \sum_{i = 0}^{m} \boldsymbol{P}_{i}^{*}b_{i,m}(t)$ with control points $\boldsymbol{P}_{i}^{*}$ ($i = 0,1, \ldots,m$), the continuity conditions $\mathrm{G}^{1}$ and $\mathrm{G}^{2}$ for Q-Bézier curves are shown by the following equations.

### Smooth continuity conditions of $\mathrm{G}^{1}$ for Q-Bézier curves

#### Theorem 3

*For two adjacent Q*-*Bézier curves* $\boldsymbol{r}_{1}(t)$ *and* $\boldsymbol{r}_{2}(t)$, *the necessary and sufficient conditions of* $\mathrm{G}^{1}$ *smooth continuity at the common joint are given by*

13 $$ \textstyle\begin{cases} \boldsymbol{P}_{0}^{*} = \boldsymbol{P}_{n} , \\ \boldsymbol{P}_{1}^{*} = [1 + \frac{n + \lambda_{n}}{\alpha (m + \lambda_{1}^{*})}]\boldsymbol{P}_{n} - \frac{n + \lambda_{n}}{\alpha (m + \lambda_{1}^{*})}\boldsymbol{P}_{n - 1} , \end{cases} $$

*where* $\alpha > 0$ *is a constant*.

#### Proof

If Q-Bézier curves $\boldsymbol{r}_{1}(t)$ and $\boldsymbol{r}_{2}(t)$ need to reach $\mathrm{G}^{1}$ continuity, they are required to reach $\mathrm{G}^{0}$ continuity at the common joint first, which means combining the end of $\boldsymbol{r}_{1}(t)$ with the beginning of $\boldsymbol{r}_{2}(t)$, that is,

14 $$ \boldsymbol{P}_{n} = \boldsymbol{r}_{1}(1) = \boldsymbol{r}_{2}(0) = \boldsymbol{P}_{0}^{*}. $$

Also, they should satisfy the same tangent direction at the joint, which means

$$\boldsymbol{r}'_{1}(1) = \alpha \boldsymbol{r}'_{2}(0), \quad \alpha > 0. $$

According to the terminal properties (6) of the Q-Bézier curve, the above equation can be simplified to

15 $$ (n + \lambda_{n}) (\boldsymbol{P}_{n} - \boldsymbol{P}_{n - 1}) = \alpha \bigl(m + \lambda_{1}^{*} \bigr) \bigl(\boldsymbol{P}_{1}^{*} - \boldsymbol{P}_{0}^{*} \bigr). $$

By combining with (14), (15) can be expressed in the form of (13), thus proving Theorem 3. □

The geometric significance of $\mathrm{G}^{1}$ continuity for two Q-Bézier curves at the joint is that the control points $\boldsymbol{P}_{n - 1}$, $\boldsymbol{P}_{n}$ ($= \boldsymbol{P}_{0}^{*}$) and $\boldsymbol{P}_{1}^{*}$ should have collinear ordering when $\boldsymbol{r}_{1}(t)$ and $\boldsymbol{r}_{2}(t)$ combine.

Specifically, let $\alpha = 1$ in (13), then (13) is equal to

$$\textstyle\begin{cases} \boldsymbol{P}_{0}^{*} = \boldsymbol{P}_{n} , \\ \boldsymbol{P}_{1}^{*} = (1 + \frac{n + \lambda_{n}}{m + \lambda_{1}^{*}})\boldsymbol{P}_{n} - \frac{n + \lambda_{n}}{m + \lambda_{1}^{*}}\boldsymbol{P}_{n - 1} . \end{cases} $$

Now, the continuity conditions of $\mathrm{G}^{1}$ degrade into the corresponding $\mathrm{C}^{1}$ continuity conditions.

### Smooth continuity conditions of $\mathrm{G}^{2}$ for Q-Bézier curves

#### Theorem 4

*For two adjacent Q*-*Bézier curves* $\boldsymbol{r}_{1}(t)$ *and* $\boldsymbol{r}_{2}(t)$, *the necessary and sufficient conditions of* $\mathrm{G}^{2}$ *smooth continuity at the common joint are given by*

16 $$ \textstyle\begin{cases} \boldsymbol{P}_{0}^{*} = \boldsymbol{P}_{n}, \\ \boldsymbol{P}_{1}^{*} = [1 + \frac{n + \lambda_{n}}{\alpha (m + \lambda_{1}^{*})}]\boldsymbol{P}_{n} - \frac{n + \lambda_{n}}{\alpha (m + \lambda_{1}^{*})}\boldsymbol{P}_{n - 1}, \\ \boldsymbol{P}_{2}^{*} = \{ \frac{(n^{2} - n + 2n\lambda_{n}) - \alpha^{2}m(m - 1) - 2m\alpha^{2}\lambda_{1}^{*} + \gamma (m + \lambda_{1}^{*})}{\alpha^{2}(m^{2} - m + 2\lambda_{2}^{*})} \\ \hphantom{\boldsymbol{P}_{2}^{*} =}{} + \frac{2\alpha^{2}m(m - 1) + 2\alpha^{2}m\lambda_{1}^{*} + 2\alpha^{2}\lambda_{2}^{*} - \gamma (m + \lambda_{1}^{*})}{\alpha^{3}(m^{2} - m + 2\lambda_{2}^{*})(m + \lambda_{1}^{*})}[\alpha (m + \lambda_{1}^{*}) + n + \lambda_{n}] \} \boldsymbol{P}_{n} \\ \hphantom{\boldsymbol{P}_{2}^{*} =}{} - [\frac{2n(n - 1) + 2\lambda_{n - 1} + 2n\lambda_{n}}{\alpha^{2}(m^{2} - m + 2\lambda_{2}^{*})} + \frac{2\alpha^{2}m(m - 1) + 2\alpha^{2}m\lambda_{1}^{*} + 2\alpha^{2}\lambda_{2}^{*} - \gamma (m + \lambda_{1}^{*})}{\alpha^{3}(m^{2} - m + 2\lambda_{2}^{*})(m + \lambda_{1}^{*})}(n + \lambda_{n})]\boldsymbol{P}_{n - 1} \\ \hphantom{\boldsymbol{P}_{2}^{*} =}{} + [\frac{n^{2} - n + 2\lambda_{n - 1}}{\alpha^{2}(m^{2} - m + 2\lambda_{2}^{*})}]\boldsymbol{P}_{n - 2}, \end{cases} $$

*where* $\alpha > 0$ *is a constant*, *and* *γ* *is an arbitrary constant*.

#### Proof

If Q-Bézier curves $\boldsymbol{r}_{1}(t)$ and $\boldsymbol{r}_{2}(t)$ reach $\mathrm{G}^{2}$ continuity, they are required to reach $\mathrm{G}^{1}$ continuity at the common joint first, which means

17 $$ \textstyle\begin{cases} \boldsymbol{P}_{n} = \boldsymbol{r}_{1}(1) = \boldsymbol{r}_{2}(0) = \boldsymbol{P}_{0}^{*} ,\\ \boldsymbol{r}'_{1}(1) = \alpha \boldsymbol{r}'_{2}(0),\quad \alpha > 0 , \end{cases} $$

where the value of *α* is the same as that in (13). Suppose that the vice-normal vector is $D_{1}$ for $\boldsymbol{r}_{1}(t)$ at $t = 1$ and $D_{2}$ for $\boldsymbol{r}_{2}(t)$, then we have

18 $$ \textstyle\begin{cases} D_{1} = \boldsymbol{r}'_{1}(1) \times \boldsymbol{r}''_{1}(1), \\ D_{2} = \boldsymbol{r}'_{2}(0) \times \boldsymbol{r}''_{2}(0). \end{cases} $$

Then the $\mathrm{G}^{2}$ continuity required for the vice-normal vector of $\boldsymbol{r}_{1}(t)$ and $\boldsymbol{r}_{2}(t)$ has the same direction at the joint. Combining (17) with (18), we obtain the four vectors $\boldsymbol{r}'_{1}(1)$, $\boldsymbol{r}''_{1}(1)$, $\boldsymbol{r}'_{2}(0)$, and $\boldsymbol{r}''_{2}(0)$ which are coplanar. Thus, using (17) we can obtain

19 $$ \boldsymbol{r}''_{1}(1) = \beta \boldsymbol{r}''_{2}(0) + \gamma \boldsymbol{r}'_{2}(0), $$

where $\beta > 0$ is an arbitrary constant.

If the curvatures are $\kappa_{1}(1)$ and $\kappa_{2}(0)$ for the curves of $\boldsymbol{r}_{1}(t)$ and $\boldsymbol{r}_{2}(t)$, respectively, we obtain

20 $$ \textstyle\begin{cases} \kappa_{1}(1) = \frac{ \vert \boldsymbol{r}'_{1}(1) \times \boldsymbol{r}''_{1}(0) \vert }{ \vert \boldsymbol{r}'_{1}(1) \vert ^{3}}, \\ \kappa_{2}(0) = \frac{ \vert \boldsymbol{r}'_{2}(0) \times \boldsymbol{r}''_{2}(0) \vert }{ \vert \boldsymbol{r}'_{2}(0) \vert ^{3}}. \end{cases} $$

As $\mathrm{G}^{2}$ continuity is required, the curvatures of $\kappa_{1}(1)$ and $\kappa_{2}(0)$ have the same value at the joint, i.e., $\kappa_{1}(1) = \kappa_{2}(0)$. Substituting (17) and (19) into (20), we have

21 $$ \begin{aligned}[b] \kappa_{1}(1) &= \frac{ \vert \boldsymbol{r}'_{1}(1) \times \boldsymbol{r}''_{1}(0) \vert }{ \vert \boldsymbol{r}'_{1}(1) \vert ^{3}} \\ &= \frac{ \vert \alpha \boldsymbol{r}'_{2}(0) \times [\beta \boldsymbol{r}''_{2}(0) + \gamma \boldsymbol{r}'_{2}(0)] \vert }{\alpha^{3} \vert \boldsymbol{r}'_{2}(0) \vert ^{3}} \\ &= \frac{\beta \vert \boldsymbol{r}'_{2}(0) \times \boldsymbol{r}''_{2}(0) \vert }{\alpha^{2} \vert \boldsymbol{r}'_{2}(0) \vert ^{3}} = \kappa_{2}(0). \end{aligned} $$

Using (21), we can see that $\beta = \alpha^{2}$. Putting the value of *β* into (19), we obtain

22 $$ \boldsymbol{r}''_{1}(1) = \alpha^{2} \boldsymbol{r}''_{2}(0) + \gamma \boldsymbol{r}'_{2}(0), $$

where the value of *α* is equal to that in (13).

From the terminal properties of the Q-Bézier curve given in Theorem 1, we can calculate the tangent vector for $\boldsymbol{r}_{1}(t)$ and $\boldsymbol{r}_{2}(t)$, which substituted into (19) yields

23 $$\begin{aligned} \boldsymbol{P}_{2}^{*} =& \biggl\{ \frac{(n^{2} - n + 2n\lambda_{n}) - \alpha^{2}m(m - 1) - 2m\alpha^{2}\lambda_{1}^{*} + \gamma (m + \lambda_{1}^{*})}{\alpha^{2}(m^{2} - m + 2\lambda_{2}^{*})} \\ &{} + \frac{2\alpha^{2}m(m - 1) + 2\alpha^{2}m\lambda_{1}^{*} + 2\alpha^{2}\lambda_{2}^{*} - \gamma (m + \lambda_{1}^{*})}{\alpha^{3}(m^{2} - m + 2\lambda_{2}^{*})(m + \lambda_{1}^{*})} \bigl[\alpha \bigl(m + \lambda_{1}^{*} \bigr) + n + \lambda_{n} \bigr] \biggr\} \boldsymbol{P}_{n} \\ &{} - \biggl[ \frac{2n(n - 1) + 2\lambda_{n - 1} + 2n\lambda_{n}}{\alpha^{2}(m^{2} - m + 2\lambda_{2}^{*})} \\ &{}+ \frac{2\alpha^{2}m(m - 1) + 2\alpha^{2}m\lambda_{1}^{*} + 2\alpha^{2}\lambda_{2}^{*} - \gamma (m + \lambda_{1}^{*})}{\alpha^{3}(m^{2} - m + 2\lambda_{2}^{*})(m + \lambda_{1}^{*})}(n + \lambda_{n}) \biggr]\boldsymbol{P}_{n - 1} \\ &{} + \biggl[ \frac{n^{2} - n + 2\lambda_{n - 1}}{\alpha^{2}(m^{2} - m + 2\lambda_{2}^{*})} \biggr]\boldsymbol{P}_{n - 2}. \end{aligned}$$

In conclusion, if the two Q-Bézier curves satisfy (13) and (23) simultaneously, then they reach $\mathrm{G}^{2}$ smooth continuity at the joint, thus proving Theorem 4. □

In particular, let $\alpha = 1$, $\gamma = 0$ in (16). Then (16) becomes

24 $$ \textstyle\begin{cases} \boldsymbol{P}_{0}^{*} = \boldsymbol{P}_{n}, \\ \boldsymbol{P}_{1}^{*} = [ 1 + \frac{n + \lambda_{n}}{m + \lambda_{1}^{*}} ]\boldsymbol{P}_{n} - \frac{n + \lambda_{n}}{m + \lambda_{1}^{*}}\boldsymbol{P}_{n - 1}, \\ \boldsymbol{P}_{2}^{*} = \{ \frac{(n^{2} - n + 2n\lambda_{n}) - m(m - 1) - 2m\lambda_{1}^{*}}{m^{2} - m + 2\lambda_{2}^{*}} \\ \hphantom{\boldsymbol{P}_{2}^{*} =}{} + \frac{2m(m - 1) + 2m\lambda_{1}^{*} + 2\lambda_{2}^{*}}{(m^{2} - m + 2\lambda_{2}^{*})(m + \lambda_{1}^{*})}(m + \lambda_{1}^{*} + n + \lambda_{n}) \} \boldsymbol{P}_{n} \\ \hphantom{\boldsymbol{P}_{2}^{*} =}{} - [ \frac{2n(n - 1) + 2\lambda_{n - 1} + 2n\lambda_{n}}{m^{2} - m + 2\lambda_{2}^{*}} + \frac{2m(m - 1) + 2m\lambda_{1}^{*} + 2\lambda_{2}^{*}}{(m^{2} - m + 2\lambda_{2}^{*})(m + \lambda_{1}^{*})}(n + \lambda_{n}) ]\boldsymbol{P}_{n - 1} \\ \hphantom{\boldsymbol{P}_{2}^{*} =}{} + [ \frac{n^{2} - n + 2\lambda_{n - 1}}{m^{2} - m + 2\lambda_{2}^{*}} ]\boldsymbol{P}_{n - 2}. \end{cases} $$

This describes the smooth continuity conditions of $\mathrm{C}^{2}$ for Q-Bézier curves. Note that the $\mathrm{C}^{2}$ smooth continuity conditions in [20] are incorrect because of an error in the second-order terminal properties given in [20]. This was because equation (5.9) in [20] was incorrectly stated.

## Steps and examples of smooth continuity for Q-Bézier curves

Using the smooth continuity conditions between Q-Bézier curves and combining with the flexible shape adjustability of these curves, we now take $\mathrm{G}^{2}$ smooth continuity as an example to discuss the basic steps of smooth continuity between Q-Bézier curves.

According to the proof of Theorem 3, the steps for smooth continuity for two Q-Bézier curves are given by: ① for any degree *n*, with shape parameters $\lambda_{i}$ ($i = 1,2, \ldots,n$) and control points $\boldsymbol{P}_{i}$ ($i = 0,1, \ldots,n$) of the initial curve $\boldsymbol{r}_{1}(t)$, then ② let $\boldsymbol{P}_{n} = \boldsymbol{P}_{0}^{*}$ so that $\boldsymbol{r}_{1}(t)$ and $\boldsymbol{r}_{2}(t)$ have a common control point, which makes the curves reach $\mathrm{G}^{0}$ continuity; ③ given the degree *m* and shape parameters $\lambda_{i}^{ *}$ ($i = 1,2, \ldots,m$) of $\boldsymbol{r}_{2}(t)$, as well as constant $\alpha > 0$, according to the second equation in (16), calculate the second control point $\boldsymbol{P}_{1}^{*}$ of $\boldsymbol{r}_{2}(t)$. ④ On the basis of steps ② and ③, given an arbitrary constant *γ*, using the third equation in (16), calculate the third control point $\boldsymbol{P}_{1}^{*}$ of $\boldsymbol{r}_{2}(t)$. ⑤ Given the remaining $m-2$ control points $\boldsymbol{P}_{i}^{*}$ ($i = 3,4, \ldots,m$) of $\boldsymbol{r}_{2}(t)$, then we can achieve $\mathrm{G}^{2}$ smooth continuity between two adjacent Q-Bézier curves.

Obviously, repeating the above smooth continuity steps can achieve $\mathrm{G}^{2}$ smooth continuity between multiple Q-Bézier curves. A similar process can be used to obtain the steps for $\mathrm{G}^{1}$ smooth continuity.

### Example 1

**Figure 2:**
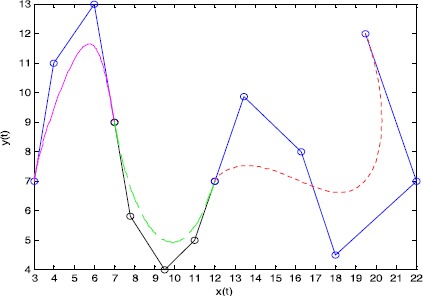
$\pmb{\mathrm{G}^{1}}$ **smooth continuity of Q-Bézier curves.**

Figure 2 shows $\mathrm{G}^{1}$ smooth continuity of a cubic curve, a quartic curve, and a quintic curve from left to right. The first example shows the joining of a cubic curve and a quartic curve, where the shape parameters are $(\lambda_{1},\lambda_{2},\lambda_{3}) = ( - 1,2,1)$ and $(\lambda_{1},\lambda_{2},\lambda_{3},\lambda_{4}) = (1,2, - 1,1)$, respectively, as well as a scale factor of $\alpha = 1$. The second example is a quartic Q-Bézier curve spliced with a quintic Q-Bézier curve, where the shape parameters are $(\lambda_{1},\lambda_{2},\lambda_{3},\lambda_{4},\lambda_{5}) = ( - 4, - 1,1,2,1)$ for the quintic Q-Bézier curve, with a scale factor of $\alpha = 7/2$. The broken lines indicate the control polygons of the Q-Bézier curves; the circular points indicate control points for the curve.

### Example 2

**Figure 3:**
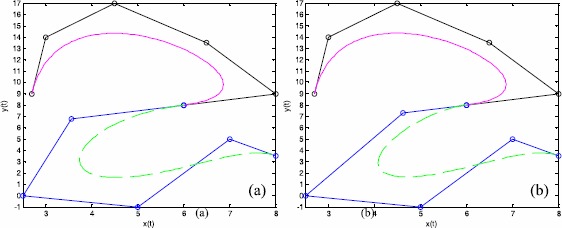
$\pmb{\mathrm{G}^{1}}$ **smooth continuity of quintic Q-Bézier curves.**

Figure 3 shows an example of ‘Type 2’ modeling, based on the $\mathrm{G}^{1}$ smooth continuity conditions between two quintic Q-Bézier curves. The shape parameters for the top and bottom curves are $(\lambda_{1},\lambda_{2},\lambda_{3},\lambda_{4},\lambda_{5}) = (1, - 1, - 2, 1, 0.5)$ and $(\lambda_{1},\lambda_{2},\lambda_{3},\lambda_{4},\lambda_{5}) = (1, 2, - 1, 0.5, 1)$, respectively. The scale factors in Figures 3(a) and 3(b) are $\alpha =3/4$ and $\alpha = 4/3$, respectively. Here, the broken lines and circular points in Figure 3 indicate the same features as in Figure 2. As can be seen from Figure 3, the value of the scale factor *α* for various $\mathrm{G}^{1}$ continuity conditions can alter the position of the second control point of the bottom curve, thus changing the bottom curve’s shape.

### Example 3

**Figure 4:**
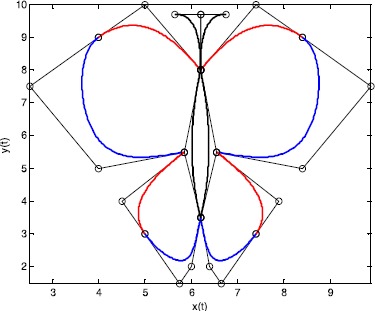
**Example of a butterfly curve.**

Figure 4 shows an example of a butterfly curve using the $\mathrm{G}^{1}$ continuity conditions. The butterfly curve is constructed using eight quadric Q-Bézier curves and four cubic Q-Bézier curves based on the $\mathrm{G}^{1}$ continuity conditions, with the curves at the joints marked with different colors. The broken lines and circular points in Figure 4 indicate the same features as in Figure 2.

### Example 4

**Figure 5:**
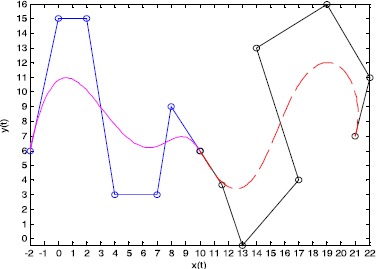
**Example of** $\pmb{\mathrm{G}^{2}}$ **smooth continuity.**

Figure 5 illustrates $\mathrm{G}^{2}$ smooth continuity of a sextic Q-Bézier curve and a septic Q-Bézier curve with scale factors $\alpha = 3/2$ and $\gamma = 1/3$, as well as shape parameters $(\lambda_{1},\lambda_{2},\lambda_{3},\lambda_{4},\lambda_{5},\lambda_{6}) = ( - 1, - 1,1.5, - 1, - 2,1)$ and $(\lambda_{1},\lambda_{2},\lambda_{3},\lambda_{4},\lambda_{5},\lambda_{6},\lambda_{7}) = ( - 1,2,1,2.5, - 1, 0,1)$. The broken lines and circular points in Figure 5 indicate the same features as in Figure 2. Figure 5 shows that the splicing curves are smooth and natural at the common joint.

### Example 5

**Figure 6:**
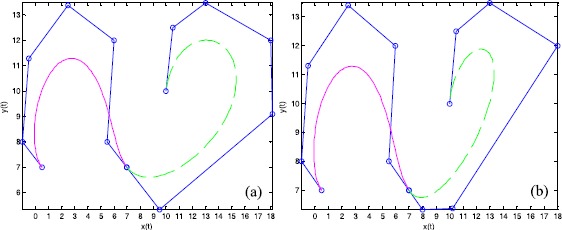
$\pmb{\mathrm{G}^{2}}$ **smooth continuity of sextic Q-Bézier curves.**

Figure 6 shows $\mathrm{G}^{2}$ smooth continuity of two sextic Q-Bézier curves. The shape parameters are $(\lambda_{1},\lambda_{2},\lambda_{3},\lambda_{4},\lambda_{5},\lambda_{6}) = ( - 1, - 1, 1.5, - 1, - 2, 1)$ and $(\lambda_{1},\lambda_{2},\lambda_{3},\lambda_{4},\lambda_{5}, \lambda_{6}) = (1, 1.5, - 1, 2, 1, - 1.5)$ from left to right in Figure 6. The scale factors are $\alpha = 3/5$ and $\gamma = 2/3$ in Figure 6(a), and $\alpha = 3/2$ and $\gamma = 3/4$ in Figure 6(b). The broken lines and circular points in Figure 6 indicate the same features as in Figure 2. Figure 6 shows that the second and third control points of the second curve in the splicing curves are altered by changing the value of two scale factors, thus changing the shape of the second curve.

## Shape adjustment of the smooth continuity between Q-Bézier curves

Compared to classical Bézier curves, Q-Bézier curves have multiple shape parameters, allowing adjustment of the local or global shape. However, altering the control points at the same time as the shape parameters does not affect the smoothness of the curve. In this paper, we will now examine the issue of shape adjustment of $\mathrm{G}^{1}$ and $\mathrm{G}^{2}$ continuity using, as an example, the smooth continuity between two Q-Bézier curves. A similar argument can be applied to multiple curves.

### Proposition 1

*In the case where the control points and* $\mathrm{G}^{1}$ *continuity for the splicing curves are not changed*, *we can adjust the local and global shape of the splicing curves*.

### Proof

From Theorem 2, $\mathrm{G}^{1}$ continuity only needs to have the same tangent direction at the common joint between adjacent Q-Bézier curves, but modifying any shape parameters for part of curves simply impacts on the size of the tangent vector without changing the direction. Thus, Proposition 1 is proved. □

Specifically, referring to a Q-Bézier curve with multiple shape parameters, the local shape of the splicing curves can all be modified so long as changing shape parameters. Such a property gives the Q-Bézier curves their flexible shape adjustability.

### Example 6

**Figure 7:**
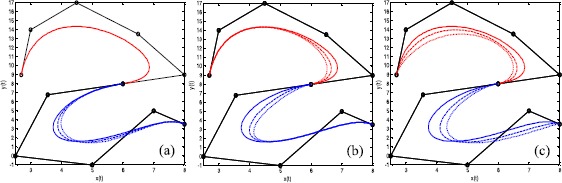
**Examples of shape adjustment of** $\pmb{\mathrm{G}^{1}}$ **smooth continuity.**

Figure 7 shows examples of local and global shape adjustment of $\mathrm{G}^{1}$ smooth continuity for the splicing curves shown in Figure 3(a). The solid lines represent the initial curves, with dashed lines and dotted lines showing the modified curves. The broken lines and circular points in Figure 7 indicate the same features as in Figure 2. Figure 7(a) shows local shape adjustment by altering one shape parameter of the second curve. It can be seen from Figure 7(a) that the shape of a part of one curve is affected by changing a single shape parameter. Figure 7(b) shows local shape adjustment through modification of the shape parameters of the $\mathrm{G}^{1}$ smooth continuity conditions. Figure 7(c) shows global shape adjustment by changing the shape parameters of two splicing curves. This example shows the flexible shape adjustability of $\mathrm{G}^{1}$ smooth continuity of the splicing curves.

Similarly, we can prove the following proposition.

### Proposition 2

*Based on* $\mathrm{G}^{2}$ *smooth continuity of the splicing curves*, *the following conclusions can be reached*: *① If the control points and* $\mathrm{G}^{2}$ *continuity for all the splicing curves are not changed*, *we can adjust the local shape of the splicing curves by altering shape parameters*. *② If* $\mathrm{G}^{2}$ *smooth continuity is unchanged*, *then global shape adjustment of the splicing curves can be achieved by altering shape parameters and control points*.

### Example 7

**Figure 8:**
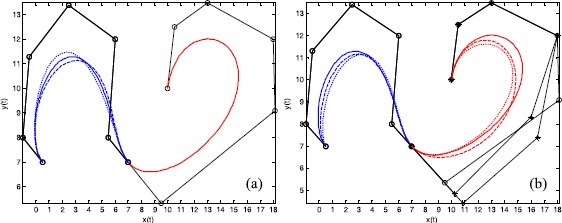
**Examples of shape adjustment of** $\pmb{\mathrm{G}^{1}}$ **smooth continuity.**

Figure 8 shows the shape adjustment of $\mathrm{G}^{2}$ smooth continuity for the splicing curves in Figure 6(a). The solid lines in Figure 8 indicate the initial curves, with the dashed lines and dotted lines representing curves with modified shape parameters. The broken lines and circular points in Figure 8 indicate the same features as in Figure 7. The asterisks indicate the modified control points. Figure 8(a) shows local shape adjustment by altering one of the shape parameters of the first curve. Figure 8(b) shows global shape adjustment by changing two control points of the second curve and the shape parameters of two sextic Q-Bézier curves.

## Conclusions

In this paper, we described the $\mathrm{G}^{1}$ and $\mathrm{G}^{2}$ smooth continuity conditions between two adjacent Q-Bézier curves of degree *n* and analyzed the influence rules of shape parameters on the shapes of splicing curves, as well as the basic steps of smooth continuity. We feel our work is significant since our proposals help to simplify the construction and computer realization of complex curves as well as extend the conclusions presented in [20]. The modeling examples show the effectiveness of the proposed methods: our proposed $\mathrm{G}^{1}$ and $\mathrm{G}^{2}$ continuity conditions for Q-Bézier curves are better than the existing continuity conditions described in [20]. The benefits and features of the proposed methods can be summarized as follows:

- Our proposed $\mathrm{G}^{1}$ and $\mathrm{G}^{2}$ continuity conditions for Q-Bézier curves of degree *n* extend the conclusions about the continuity condition given in [20].
- For a piecewise generalized Q-Bézier curve with $\mathrm{G}^{1}$ or $\mathrm{G}^{2}$ smooth continuity, we can adjust its global and local shape by changing the shape parameters.
- The continuity conditions proposed in this paper are not only intuitive and easy to implement, but also offer more degrees of freedom for the construction of complex curves used in engineering design.

It is worth noting that the proposed methods in this paper are the first to consider the $\mathrm{G}^{1}$ and $\mathrm{G}^{2}$ geometric continuity conditions for Q-Bézier curves.
